# Most Efficient Treatment of Scalp Wounds

**Published:** 1884-05

**Authors:** J. McF. Gaston

**Affiliations:** Atlanta, Ga.


### MOST EFFICIENT TREATMENT OF SCALP WOUNDS.

BY J. McF. GASTON, M. D., ATLANTA, GA.

The time-honored impression that the air should be excluded from wounds of all kinds has latterly sought a solution in the demonstration of the ingress of minute organisms from the atmosphere, which is claimed to supply the *materies morbi*, or at least to aggravate the operations of any and all other causes of trouble to the organization. How far complications may arise from this source remains to be settled; and in the meantime some common sense is requisite in the practical application of means of relief for the ordinary lesions of the various tissues of the body without regard to the preconceived views of speculators and experimentalists.

As a contribution to this class of observations I have to submit the results of a considerable experience in the treatment of wounds of the scalp, extending to the bony structure of the cranium.

If it is understood at the outset that I accept the doctrine of closing hermetically all clean incised wounds, whether deep-seated or superficial, in whatever order of tissues they may be found, so as to effect a union by adhesive inflammation, it must be based upon the recognition that there are no injuries within that may require an outlet. The exudations from lacerated or bruised tissues cannot safely be pent up by the closure of the superficial parts, and whenever this result is anticipated the closure of external wounds cannot secure a satisfactory issue to the case.

In the lesions of the scalp, even by cutting instruments that divide the periosteum, and involve the external laminæ of the bone, it is, to say the least, questionable as to the immediate closure of the wound; and in those injuries which are most frequent, from blows inflicted by falling upon angular bodies, or by the stroke of a cudgel, whether of wood or metal, the subjacent modification is such as to require relief primarily to the serous exudation and, secondly, to the purulent discharge, so that an outlet is essential in their treatment.

The wide field of investigation as to drainage becomes involved in the decision of the proper management of this class of cases, and the underlying principle of the practical question is embodied in the decision of the indication presented by the state of things within, for an outlet. Whatever may be the nature of the injury, or the character of the surgical operation implicating the subcutaneous tissues, we must determine in advance as to this point; and unless there is an assurance amounting almost to certainty, that no serous exudation or purulent collection will ensue, the external opening should not be completely closed. If the tissues within are liable to undergo decomposition, the way must be left open for the discharge.

I am fully aware that the fundamental issue lies in the greater or less predisposition to such degeneration from the wound being open or closed, and that those who claim a germinal element for the atmospheric contamination seek to prevent this by exclusion; yet it is evident that with all the precautions that may be adopted for this end it is impossible to obviate the disintegrating process within for all cases. Cleanse a wound completely from the bottom of all coagula, and use every antiseptic application available on the occasion of making the dressing, yet if there are contused tissues beneath the surface, no external measures can avail to prevent the trouble within, and the decomposed fluids must be allowed to escape if we would avoid further disturbance.

Wounds of the scalp were originally treated by me with adhesive plaster, under the supposition that stitches were liable to induce erysipelas or other consequences of the local irritation; but I soon found that by the accurate apposition of the margins a speedy superficial agglutination occurred, while the parts beneath were not in a healthy state. A few cases of constitutional disturbance growing out of this degeneration beneath the hairy investment, with the urgent local indications for opening up the wound for the discharge of pus that had formed in contact with the periosteum or immediately upon the bone of the cranium, convinced me that this shutting up of a contused wound was not a safe proceeding.

In view of the certain dangers encountered by the union of the edges with the adhesive plaster, I was inclined to risk the lesser evils likely to attend the sutures, and resorted to them with water dressings, hoping thus to favor the escape of any grumous blood that might remain in the bottom of the wound, and to permit the outlet of the serous exudation from the lacerated margins of the wound. With only so many stitches as seemed requisite to keep the parts in apposition, there was still a great proclivity to speedy union of the superficial tissues of the scalp, and though no erysipelas ever appeared as the consequence of suturing the scalp, there were untoward effects from the closing of the wound, such as had been encountered from the close approximation by the adhesive plaster. I hoped that this difficulty would be effectually obviated by the abandonment of strips and stitches with the substitution of interlacing of the hair so as to bring the edges together.

This mode of proceeding had been recommended in the periodical literature of that period, but to whom it should be credited is not recalled, and it seemed to fulfill the indications for an approximation of the divided tissues without presenting any obstacle to the discharges, so that the results were more satisfactory than with stitches or plaster. In the meantime observing that with all the care I could use in weaving the small tufts of hair across from side to side alternately throughout the extent of the wound, and securing them with a bandage at each interlacing, all would be loosened when I went to repeat the dressing so as to afford no traction upon the margins, it struck me that no real benefit accrued from this process. Then it was, for the first time, that I was brought to the conclusion that the wounds of the hairy envelope of the cranium did not require any traction to retain their margins in apposition, and that I might dispense with such appliances altogether. From that time forward I have adopted the plan of clipping away the hair for an area that admitted of the application of a simple lint compress, moistened with water, or with the addition of tincture of arnica, and occasionally, for the relief of pain or soreness, adding some laudanum. The medication has been varied, and after a week when there may be some suppuration, has been modified by smearing the lint with a camphorated resin ointment, and with this the cure has uniformly progressed favorably, leaving no broader cicatrix than had followed the other measures.

As it may appear to some that my good results with this simple means of treatment are owing to the absence of complications in the cases, I may state that my field of observation in this class of injuries during the past sixteen years has been most ample, presenting the most varied and most aggravated specimens of contused scalp wounds. Among others I may note the case of an athletic man who had received many strokes from a bludgeon that opened eleven wounds through the scalp so as to expose the cranium, with sundry other cuts and contusions on different parts of the head. In some places these incisions made angles with each other, as his position was shifted towards the two antagonists who attacked him, and the corners were even elevated and displaced, so that it was a matter of doubt whether they could be retained in place without stitches. But in view of the wretched plight of the sufferer I was indisposed to add to his pain by using a needle; and washing the wounds thoroughly with carbolized water, so as to get clear of all coagula, the co-optation of the flaps and angles was ultimately effected satisfactorily, and the lint compress covering nearly all of the head, was saturated with a camphorated solution of carbolic acid, with a moderately firm bandage securing the whole. The oozing of blood from the numerous incisions required frequent changes of the dressing during the first afternoon, but on the following day the same lint compress was kept on and constantly moistened with the ten per cent carbolic camphorated solution. This course was continued for a week, and subsequently the camphorated resin salve was applied with the lint. Strange to say there was very slight suppuration from any of the wounds, and in the course of fifteen days most of them were healed. The profuse hemorrhage that occurred at the outset from such numerous wounds involving so large a portion of the scalp afforded exemption from brain trouble, subsequently during the fever of reaction, and there was very slight indication of mental perturbation at any time during the treatment.

Having had frequent opportunities to note the effects of blows upon the head, I have been impressed with the fact that concussion of the brain ard extravasation within the cranium occur in those cases of incision of the scalp more rarely than in violence that does not produce a lesion of the tissues. Two notable instances under my observation illustrate the serious results of bloodless injuries. One being a blow upon the side of the head in which there was not even abrasion of the scalp that caused death, and the post mortem revealed effusion of blood upon the outer surface of the left lobe of the brain. The other, in which a man fell backwards upon his occipital protuberance, causing fracture of the base of the skull, from which he died, and yet the scalp gave no signs of being even bruised. It would appear that the propagation of shock to the brain must be diminished by the division of the integument of the head, and secondarily the flow of blood from the wound lessens the liability to suffer from inflammatory action. Hence the great importance of allowing all the exudation to escape during the early management of such injuries, and the necessity for evacuation of any pus that may be formed in contact with the cranium in the further progress of the treatment.

There are few surgeons who, with all the antiseptic outfit of the present day at hand, would undertake to close up a wound in any part that was known to contain mangled tissues that must be detached by disintegration at an early or later period of the process of reparation, and in these lacerated and bruised conditions of the pericraneal integument, the same precaution is requisite in leaving an outlet.

It is not claimed that in every instance of wounds of the scalp unfavorable results do ensue from immediate closure of the incision, but I do insist that it is the safest and best treatment to dispense with adhesive plaster, stitches, interlacing of the hair, or any other means of traction to approximate the edges of scalp wounds; and to apply simply a dressing of wet lint with the moderate support of a bandage at the outset, and a mild salve, after a few days have elapsed, if there is suppuration. The firm consistence of the scalp keeps the margins in apposition, and with the internal bony support and the external compress of lint, the lightly applied bandage keeps the subjacent parts drained effectually, so that the adhesive inflammation proceeds under the most advantageous conditions for uniting and healing the tissues. It might be supposed that a broader cicatrix results in cases treated thus than in others, but I have not found it so, and with a profound conviction that the initiation, progress, and termination of this simple mode of treating scalp wounds is the most satisfactory, it is heartily and confidently recommended to the adoption of the profession.

